# Social network properties predict chronic aggression in commercial pig systems

**DOI:** 10.1371/journal.pone.0205122

**Published:** 2018-10-04

**Authors:** Simone Foister, Andrea Doeschl-Wilson, Rainer Roehe, Gareth Arnott, Laura Boyle, Simon Turner

**Affiliations:** 1 Animal & Veterinary Sciences, SRUC, Roslin Institute Building, Easter Bush, Midlothian, United Kingdom; 2 The Roslin Institute and Royal (Dick) School of Veterinary Studies, University of Edinburgh, Edinburgh, Midlothian, United Kingdom; 3 School of Biological Sciences, Queen’s University Belfast, Northern Ireland, United Kingdom; 4 Teagasc, Pig Development Department, Animal & Grassland Research and Innovation Centre, Moorepark, Fermoy, Co. Cork, Ireland; Arizona State University & Santa Fe Institute, UNITED STATES

## Abstract

Post-mixing aggression in pigs is a harmful and costly behaviour which negatively impacts both animal welfare and farm efficiency. There is vast unexplained variation in the amount of acute and chronic aggression that dyadic behaviours do not fully explain. This study hypothesised that certain pen-level network properties may improve prediction of lesion outcomes due to the incorporation of indirect social interactions that are not captured by dyadic traits. Utilising current SNA theory, we investigate whether pen-level network properties affect the number of aggression-related injuries at 24 hours and 3 weeks post-mixing (24hr-PM and 3wk-PM). Furthermore we compare the predictive value of network properties to conventional dyadic traits. A total of 78 pens were video recorded for 24hr post-mixing. Each aggressive interaction that occurred during this time period was used to construct the pen-level networks. The relationships between network properties at 24hr and the pen level injuries at 24hr-PM and 3wk-PM were analysed using mixed models and verified using permutation tests. The results revealed that network properties at 24hr could predict long term aggression (3wk-PM) better than dyadic traits. Specifically, large clique formation in the first 24hr-PM predicted fewer injuries at 3wk-PM and high betweenness centralisation at 24hr-PM predicted increased rates of injury at 3wk-PM. This study demonstrates that network properties present during the first 24hr-PM have predictive value for chronic aggression, and have potential to allow identification and intervention for at risk groups.

## Introduction

Post-mixing aggression occurs as a means of establishing a social hierarchy amongst unfamiliar conspecifics [1]. In commercial industry, pigs are frequently regrouped as they are transferred between production stages. Regrouping starts at four weeks when piglets are weaned from the sow and moved to their weaner group (which can be mixed or single sex groups) consisting of multiple different litters. This process is repeated when pigs are moved from their weaner groups to grower and finisher housing. Mixing will occur again once animals are transported to the abattoir and kept in lairage. Whilst aggression during the first 24hr post-mixing is mostly explained by the need to establish dominance relationships, pigs also display chronic aggression associated with the maintenance of these relationships within stable groups of familiar conspecifics [1] [2].

Pig aggression is highly overt and causes injury in the form of skin lesions (hereby referred to as lesions), the location and number of which correspond to the type and duration of aggressive interactions [3]. Lesions provide a distinctive and quantifiable cost to the interaction, and a reliable outcome measure for hypothesis testing. In addition to physical injury, aggression induced stress is associated with elevated cortisol and heart rate [4,5], and compromised immunocompetence [6]. Aggression also reduces carcass and meat quality [7,8], as well as stunting growth and reducing feed efficiency [9,10].

A group’s ability to form a lasting dominance hierarchy is necessary for long term group stability, and there is evidence that engagement in aggression soon after mixing can improve productivity and reduce chronic aggression over the growing-finishing period [11]. In contrast, avoidance of aggression during the acute post-mixing phase tends to only delay aggression [12]. This suggests that there is a trade-off situation whereby aggression during the acute phase appears to be necessary in order to reduce chronic aggression, improve welfare, and maintain productivity. However, a large proportion of variation in the severity of aggression at both time points remains unexplained by the animals’ engagement in aggressive behaviours at the dyadic level. Even where cluster analysis identifies pigs that share greater than 80% similarity in dyadic behavioural traits (behaviours that describe the direct interactions that an animal has engaged in), large differences exist between these pigs in their number of chronic injuries. This suggests that a more refined approach to quantify behaviours may be necessary in order to fully understand the variation in injuries [13].

Social network analysis (SNA) has rapidly risen in popularity amongst behavioural scientists [14], as it offers the ability to capture and quantify social behaviours beyond the dyadic framework. A growing body of evidence suggests that an animals’ indirect social connections or ‘friends of friends’ [15] have important fitness consequences, highlighting the need to consider animal behaviour within its wider social context. Although the majority of SNA has been largely descriptive in nature [16,17], there has been a considerable increase in experimental and predictive use of network properties in recent years. Individual network position is an important predictor for survival in wild Barbary macaques [18] and juvenile male dolphins [19]. Most notably, network position exceeds the predictive value of dyadic traits for offspring survival in baboons [20]. Network level properties have also been found to be predictive of aggressive outbreaks [21], parasitism load, and infectious disease spread among social animals [22]. However, despite having considerable potential for improving welfare [23], application of SNA to farm animal behaviour, especially in a predictive context, is considerably underrepresented in the literature [24–26].

In this study we quantified commonly studied group-level network properties [23,27] in multiple groups of pigs *(Sus scrofa)*, with the objective of examining the hypothesis that network properties can be used to predict subsequent levels of injury resulting from aggressive interactions and account for variation that is unexplained by dyadic behaviours. Additionally, we compared the predictive value of network properties to that of conventional dyadic interactions in order to determine whether network properties (in particular, network properties that incorporate indirect social connections) are an important factor for subsequent injury rate. It is anticipated that applying social network analysis to post-mixing aggression in pigs will reveal the mechanisms by which certain groups manage to establish stable social relationships more rapidly and effectively than others.

## Method

### Data collection

The study was conducted and the video data collected on a private commercial farm in Ransta, Sweden with permission from the farm owner. The study comprised 1,170 pigs housed in single sex (intact males, castrated males and females), and single breed (705 purebred Yorkshire and 465 crossbred Yorkshire x Landrace). Analyses conducted with a larger dataset on the same farm have revealed no significant breed effects for aggressivity [28]. The groups were comprised of 15 pigs; 3 from 5 separate litters. The pigs were moved into their new social groups at 8 weeks old, creating 78 pens of 15 pigs. One pig was removed from the study due to injury, and which left one pen containing only 14 animals. There is a positive correlation between live weight and aggression [29,30], and weight asymmetry within a group can significantly affect the type of aggression and duration of fighting that occurs. Therefore, where possible, pigs of similar weight were grouped together (mean 27.6 kg (*SD* = 5.6)) to limit this effect.

Each group was video recorded for 24hr post-mixing. Using all-occurrence sampling, the details of aggressive interactions that each individual engaged in was recorded, including time, type of aggressive interaction (see ‘behaviours’), initiator, and receiver, as well as the animals’ pen identity, sex, breed, litter identity, and unique pig identification. Video analysis was conducted by three observers using time-lapse video software to record the duration of each behavioural occurrence to the nearest second. Inter-observer reliability was tested using three 1-hour samples of data and showed a significant degree of inter-observer agreement (mean *r =* 0.83, *p* < 0.001).

Lesions were counted at three intervals: before being mixed, 24hr post-mixing (24hr-PM), and 3 weeks later (3wk-PM) once the groups were assumed to be stable. Lesions were recorded in three regions of the body; anterior, central, and posterior, as these regions are associated with different aggressive behaviours. Lesions in the anterior portion of the body are predominantly associated with engaging in reciprocal fighting, and lesions to the posterior portion of the body are predominantly associated with receipt of non-reciprocal aggression (referred to as bullying in this study) [3]. Lesions were recorded immediately before mixing and were subtracted from those recorded at 24hr to estimate the number of lesions received due to the establishment of new dominance relationships.

### Behaviours

Pigs display both reciprocated fighting and unreciprocated bullying. Fighting was defined as aggression that lasted at least one second where both pigs engaged in biting, pushing, or head knocking the opponent. Bullying occurred when one pig received or delivered aggression with no observable retaliation occurring [28]. In this paper we define dyadic behavioural traits as behaviours derived from direct interactions (e.g. the amount of time the animals spent fighting; the number of fights that occurred). Dyadic behavioural traits derived from fighting and bullying behaviours are detailed in Table 1. These behaviours were selected due to being previously identified as significantly associated with lesions at both 24h-PM and 3wk-PM [12], and provided a useful benchmark with which to compare the ability of network properties to predict lesions.

**Table 1:** Description of dyadic traits.

Behaviour	Description
Mean duration of fighting and bullying.	Mean duration of each fight and bout of bullying that the focal pig was involved in.
Total fight duration.	Total duration of all fights that the focal pig was involved in.
Number of fights involved in.	Total number of reciprocal fights the focal pig was involved with, regardless of which pig initiated the attack.
Proportion injurious fights.	Proportion of time the focal pig spent in reciprocal fights engaged in what was deemed to be injurious fighting. Injurious fighting was defined as acts of aggression where bites were delivered at an approximate rate of 1 per 3s [3].
Duration of bullying given.	Duration of time spent in bullying in which the focal pig was the initiator.
Duration of bullying received.	Duration of time spent in bullying in which the focal pig was the recipient of the attack.

### Social network analysis

Networks were constructed in R (version 3.2.3) using the R package *igraph* [31]. The unit of analysis was the pen level network, providing 78 independent data points. Degree, eigenvector and betweenness centralisation values were obtained via the *centralization*.*degree*, *centr_eigen*, and *centralization*.*betweenness* functions in *igraph*.

Separate networks were constructed for fighting and bullying behaviours in order to determine whether they offered different predictive value. Networks were also constructed containing all aggressive behaviours (both fighting and bullying), which we refer to as ‘combined’.

#### Network terminology

In SNA, networks are presented as graphs, comprised of the individuals (referred to as nodes) and the line that connects two nodes (referred to as an edge). A directed edge allows the direction of the interactions (e.g. from the sender towards the receiver) to be incorporated in the network, whereas an undirected edge represents a bidirectional relationship [32]. SNA provides methods of describing networks as a whole (global measures), the substructures within a network, and the individuals’ network position (local measures). Identifying whether an individual holds an important or ‘central’ position within the network (known as individual centrality) is a commonly studied local measure. There are a number of different methods to define what constitutes an important position in a network and thus which individual is considered to be central [33]. For example, individuals that connect otherwise unconnected groups may play an important role in group cohesiveness [34].

### Network properties

The primary aim of this study was to identify pen-level network properties that explain the variation in injurious outcomes that is not explained by dyadic traits. Due to the limited information regarding the network properties that form during post-mixing aggression in pigs, the decision was made to include a selection of commonly used network measures in animal behaviour and identify traits that were most closely associated with the number of lesions using a stepwise regression (further details under ‘Statistical analysis’). A full list of network properties analysed in this study can be found in the supplementary material S1 and descriptions of each trait are detailed in S2. Measures that were relevant to the results section are discussed in detail below.

#### Centralisation

**Fig 1:**
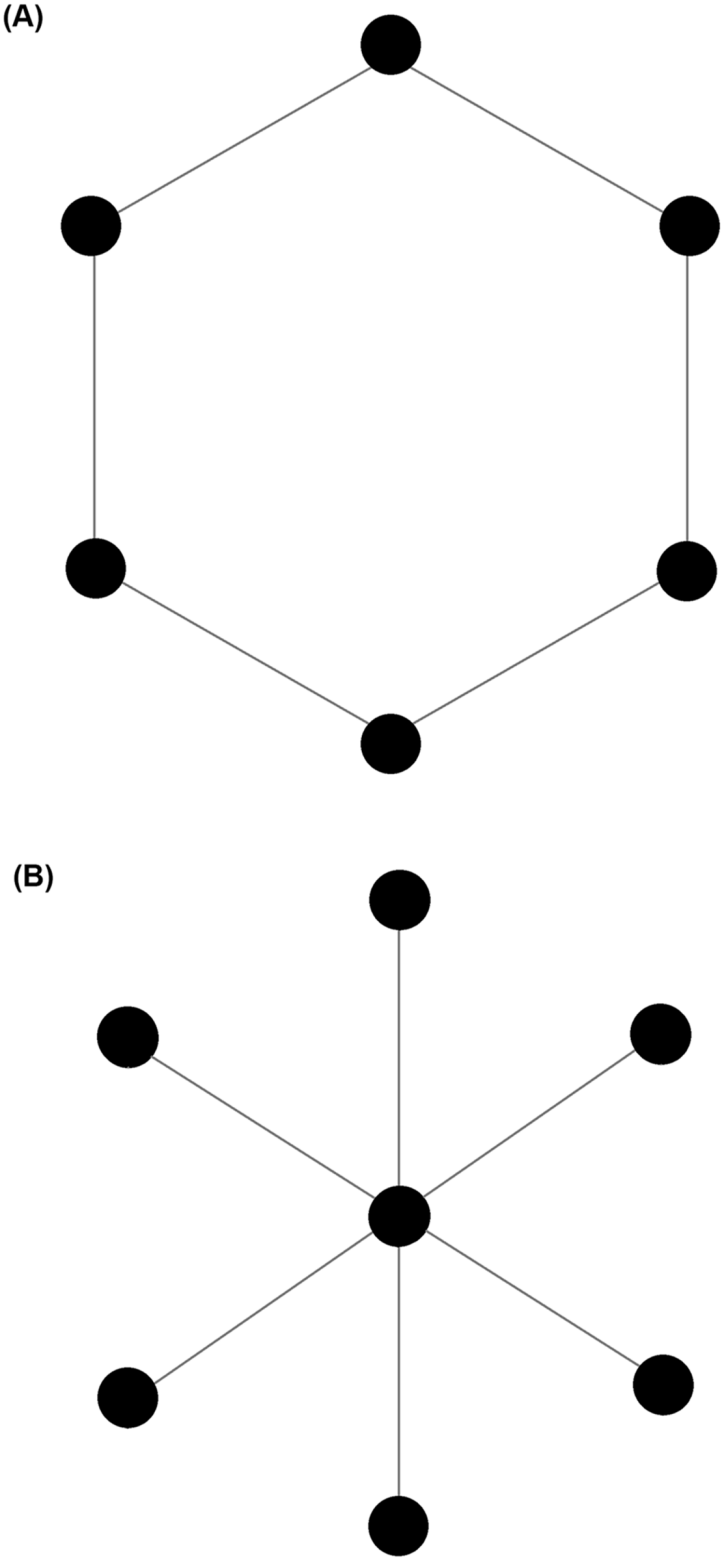
Example networks with different centralisation. A) A non-centralised ‘ring’ network. B) A maximally centralised ‘star’ network.

Freeman’s centralisation equation calculates a network level metric from individual centrality scores by summarising the disparity in centrality that exists within a network. A global value for a network (the network property) is obtained by the sum of differences in individual centrality scores between the most central animal and all other animals in the network. This sum is divided by the theoretical largest sum of differences in any network of the same size to give a value between 0 and 1, where 1 is considered a maximally centralised network [35] (further details on centralisation can found be supplementary material S1 File). Fig 1 provides an example of a non-centralised network (Fig 1A) and a highly centralised network (Fig 1B). Freeman’s equation has been successfully used to describe the structures of a number of animal social networks [24,25,36,37]. Here we describe ‘degree’, ‘betweenness’, and ‘eigenvector’ centralisation.

#### Degree centralisation

Degree centrality describes the number of direct connections an animal has. In this paper, we present three forms of degree centralisation: in-degree, out-degree and total degree. In-degree calculates how many incoming interactions an animal has (i.e. how many animals attacked the focal animal), and out-degree calculates the number of out-going interactions (i.e. how many animals the focal animal attacked) [38]. As a pen-level network property, degree centralisation describes whether certain individuals in the network either give or receive considerably more aggression than the rest of the animals in the network.

#### Betweenness centralisation

**Fig 2:**
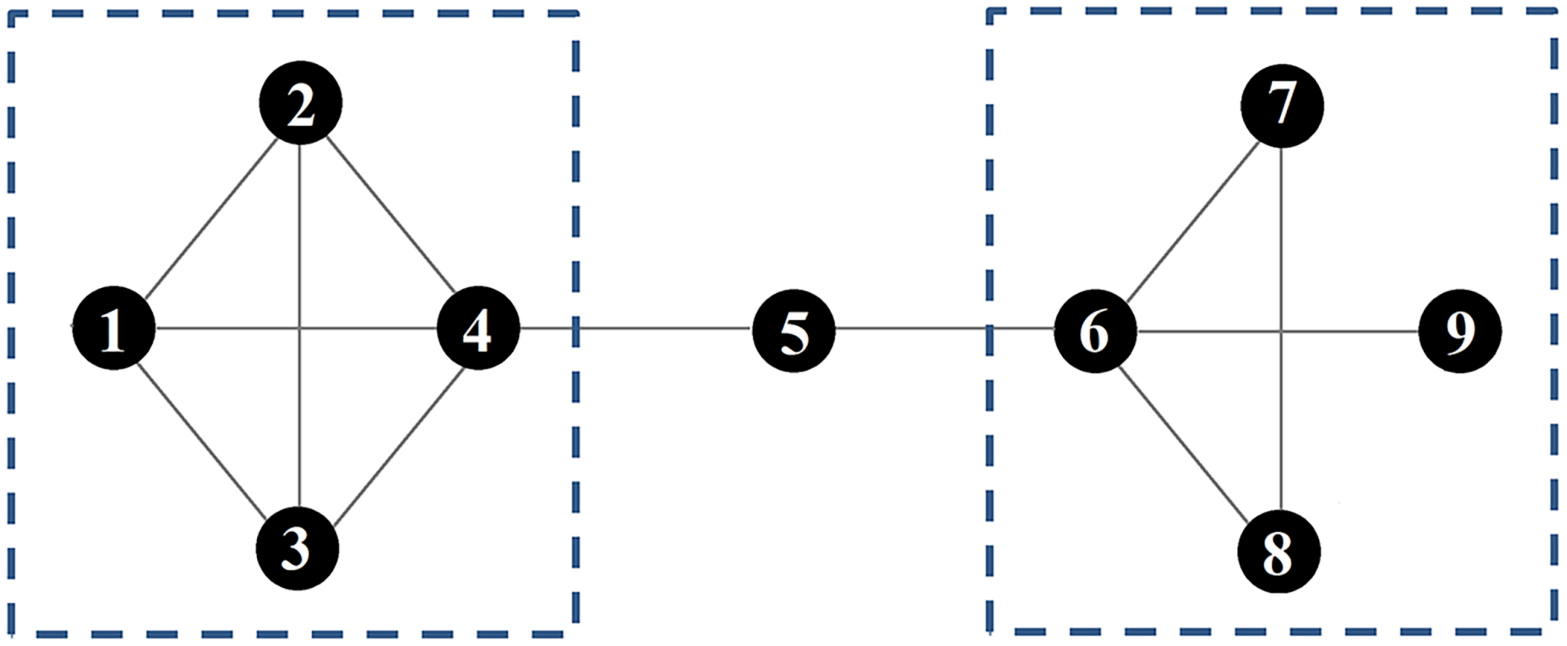
Model network. Dashed lines represent substructures (cliques).

Betweenness centrality measures the number of shortest social paths between every pair of group members in the network that pass through a particular individual. (e.g. node 5 in Fig 2 has high betweenness). In behavioural terms, networks that have high betweenness centralisation tend to contain individuals who connect other individuals that do not directly interact [39]. For example, removal of node 5 would result in the network dividing into two separate groups (Fig 2).

#### Eigenvector centralisation

At the individual level, eigenvector centrality reflects the sum of the centralities of an individual’s neighbours. An individual may achieve high centrality due to having many connections (high degree), or by interacting with individuals with a high degree, or a combination of both [40,41]. Thus eigenvector centrality extends the scope of degree centrality, by accounting for the quality and not just the quantity of connections an individual has. At a network level, a pen with high levels of eigenvector centralisation would have a small number of well-connected individuals, with the rest of the group being considerably less well connected. In terms of aggressive networks in this study, it would suggest that engagement in aggressive behaviour (both giving and receipt) is unevenly displayed within the group.

#### Size of largest clique

A clique represents a fully connected subgroup of individuals whereby each individual in the clique directly interacted with all others in the clique [32,34]. In this paper we present the size of the largest clique. In Fig 2 the nodes *1*, *2*, *3*, and *4* form the largest clique in the network. To calculate the size of the largest clique in each network, we used the *igraph* function “*clique_num”*. This function finds the largest clique in each network and returns the number of individuals that belong to this subgroup.

### Statistical analysis

All statistical analysis was carried out in SAS v9.4.

#### Identification of predictive network and dyadic traits using mixed models

Our statistical approach was designed to test the extent to which network properties accounted for the remaining variation in the pen level lesion scores once the fixed effects of breed, sex, mean body weight of the pen, and experimental batch had been accounted for. Furthermore, as the number of aggressive interactions that occur within a pen is a strong predictor of the number of lesions [12], this too was included as a fixed effect. Our statistical approach consisted of three steps. The first step was to run a mixed model with the lesion scores as the response variate and fixed effects as predictors to obtain residuals reflecting the variance in lesion scores not accounted for by the fixed effects (hereafter referred to as the ‘partial model’). The next step was to run a stepwise regression containing the lesion score residuals from the partial model as the response variate and the network and dyadic traits as the predictors in order to identify traits that best accounted for the remaining variation in lesion score residuals. The final step was to incorporate the identified network or dyadic trait into the partial model (hereafter referred to as the ‘full model’) to provide full model fit statistics. This allowed us to compare the improvement that the network properties and dyadic traits provided in contrast with the partial model.

*Partial model*. To isolate the variation in lesions not accounted for by fixed effects, the average number of lesions in each body region in the pen (calculated by summing the number of lesions in each pen and dividing by the number of animals in the pen) was entered as the response variable. The pen average was used due to the loss of one animal in a pen, which led to one pen having 14 animals rather than 15. Breed, sex, and mean number of aggressive interactions that occurred in the pen were entered as fixed effects; experimental batch was entered as a random effect, and mean body weight of the pen as a covariate. This was carried out using the SAS mixed procedure. The mixed model provided pen level lesion score residuals reflecting the remaining variance.

*Stepwise regression*. Stepwise regressions provide a method of fitting regression models by adding or removing predictor variables by an automatic procedure. Variables are either added or removed based upon the test statistics of the estimated coefficient [42]. This was carried out using the SAS regression procedure with stepwise model selection method.

To identify which network properties provided the best model fit once the fixed effects of the pen had been accounted for, the pen level lesion score residuals were entered as the response variable in a stepwise regression with all network properties (see S1 Table) as the predictors. The stepwise regression identified the network trait(s) that provided the best model fit (based on the test statistics of the estimated coefficient) to account for the remaining variance in pen level lesion score residuals. The stepwise regression was then repeated for a list of dyadic traits (see Table 1).

*Full model*. Finally, the network properties and dyadic traits that were identified via the stepwise regression to provide the best model fit were then added to the full model in order to provide full model fit statistics (RMSE, AIC, R^2^). The full model was the same as the partial model described above, with the addition of the traits that provided the best fit for the remaining variance.

Model assumptions including normality and variance inflation factors were checked and confirmed to be within the acceptable range.

### Permutations

In social network analysis permutations are routinely used in order to generate replicates to compare to the observed dataset and to account for the non-independence of the data [43]. Conventional statistics have been successfully applied in other network data studies that contained adequate replication and independent data (34,41). In this study we had 78 observed replicates, that each provided an independent network metric. Thus conventional statistics were appropriate [43] and were the primary statistical methodology utilised in this study. The use of conventional statistics was also prioritised as it allowed us to compare model fit between dyadic traits and network properties more easily than would be achieved using permutation methodology alone. An additional reason for using permutations in SNA is to evaluate whether the observed network is representative of the real network [41]. In this study we recorded and included all interactions that occurred in the 24h period of interest, which provides a high level of confidence that our networks are representative of the real network for this period of time.

However, we utilise permutation tests in this study in order to confirm the results of the mixed models and the significance of the network properties identified by the stepwise regression.

Keeping all other effects stable, the permuted network properties (betweenness centralisation and largest clique size) were entered into the full model to obtain a coefficient for the network property of interest. This was repeated 5000 times to provide a distribution of coefficients with which the coefficient from the observed network could be compared. A *p*-value was obtained by calculating the number of times the observed network coefficient was greater than the coefficients derived from the permuted networks, divided by the number of permutations. This value was deducted from 1 in the case where the observed network model provided a negative coefficient (indicating that the network property predicted a reduction in lesion scores), in order to provide an accurate *p*-value [44].

### Correlation of predictive network and dyadic traits

A Spearman rank correlation of the dyadic and network properties was performed to verify the uniqueness of traits and to avoid errors due to duplication.

### Ethical note

This study was carried out in accordance with the recommendation outlined in the European Guidelines for accommodation and care of animals and the UK Government DEFRA animal welfare codes. The work was approved by SRUC’s Animal Ethics Committee (application number ED AE 5/2005).

## Results

### Descriptive statistics

A total of 9313 aggressive interactions were recorded during the 24hr period post introduction. Animals that did not engage in aggression were still included in the networks as isolates. Fighting and bullying occurred with approximately equal frequency (mean number of fights per pen = 62.38, SD = 24; mean number of bullying interactions per pen = 57, SD = 26.4).

Despite the standardisation of resource provision and the abiotic environment in this study, considerable variation in network structure existed. As expected, there was also large variation in the amount of group level injury at 24hr-PM and 3wk-PM (see Table 2). The maximum size of a fighting or bullying clique was 7 individuals (47% of pen members).

**Table 2:** Descriptive statistics for a) network properties and b) pen level skin lesions.

a) Descriptive statistics of network properties				
Network Trait	Network type	Median	Minimum	Maximum
Degree centralisation	Fight	0.30	0.17	0.54
	Bully	0.33	0.12	0.61
	Fight & Bully	0.35	0.14	0.64
Betweenness centralisation	Fight	0.15	0.06	0.41
	Bully	0.21	0.08	0.62
	Fight & Bully	0.14	0.01	0.53
Eigenvector centralisation	Fight	0.51	0.32	0.74
	Bully	0.52	0.29	0.76
	Fight & Bully	0.4	0.16	0.60
Largest clique size	Fight	4	3	7
	Bully	4	3	7
	Fight & Bully	5	4	8
b) Descriptive statistics of pen level skin lesions				
Time (24hr-PM/3wk-PM)	Body region	Median	Minimum	Maximum
24hr-PM	Anterior	18.33	3.67	45.27
	Central	9.33	1.93	26.13
	Posterior	4.07	-19.13^a^	11.80
	Total	31.53	8.43	82.47
3wk-PM	Anterior	10.27	5.33	15.93
	Central	10.07	4.67	18.67
	Posterior	4.40	0.80	7.87
	Total	24.73	11.13	42.13

^a^ Negative lesion values resulted from some animals having lower lesions in certain body regions after mixing than before.

Further information on the characteristics of lesions can be found in Desire et al (2015) (12). Descriptive statistics for all network properties analysed in this study can be found in supplementary material S3.

### Fixed effects on number of lesions

There was a significant breed effect for skin lesions in all body regions at 3wk-PM (anterior: *F*_*1*,*59*_ = 8.13, *p* = 0.006; central: *F*_*1*,*59*_ = 8.81, *p* = 0.004; posterior: *F*_*1*,*59*_ = 5.93, *p* = 0.018; total: *F*_*1*,*59*_ = 9.74, *p* = 0.003) with pure Yorkshire having significantly higher lesions at 3wk-PM than Yorkshire Landrace crossbreeds. There was also a significant experimental batch effect on lesions at 24hr-PM (anterior: *F*_*13*, *58*_ = 2.22, *p* = 0.002; central: *F*_*13*, *59*_ = 2.14, *p* = 0.024; posterior: *F*_*13*, *54*_ = 2.68, *p* = 0.006; total: *F*_*13*, *58*_ = 3.07, *p* = 0.002) and 3wk-PM (anterior: *F*_*13*, *59*_ = 3.79, *p*<0.001; central: *F*_*13*, *59*_ = 2.16, *p* = 0.023; posterior: *F*_*13*, *59*_ = 5.17, *p*<0.001; total: *F*_*13*, *59*_ = 3.39, *p*<0.001). The number of fights per pen was not found to have a significant effect at either time point.

### Predictive value of SNA and dyadic traits on number of lesions

**Fig 3:**
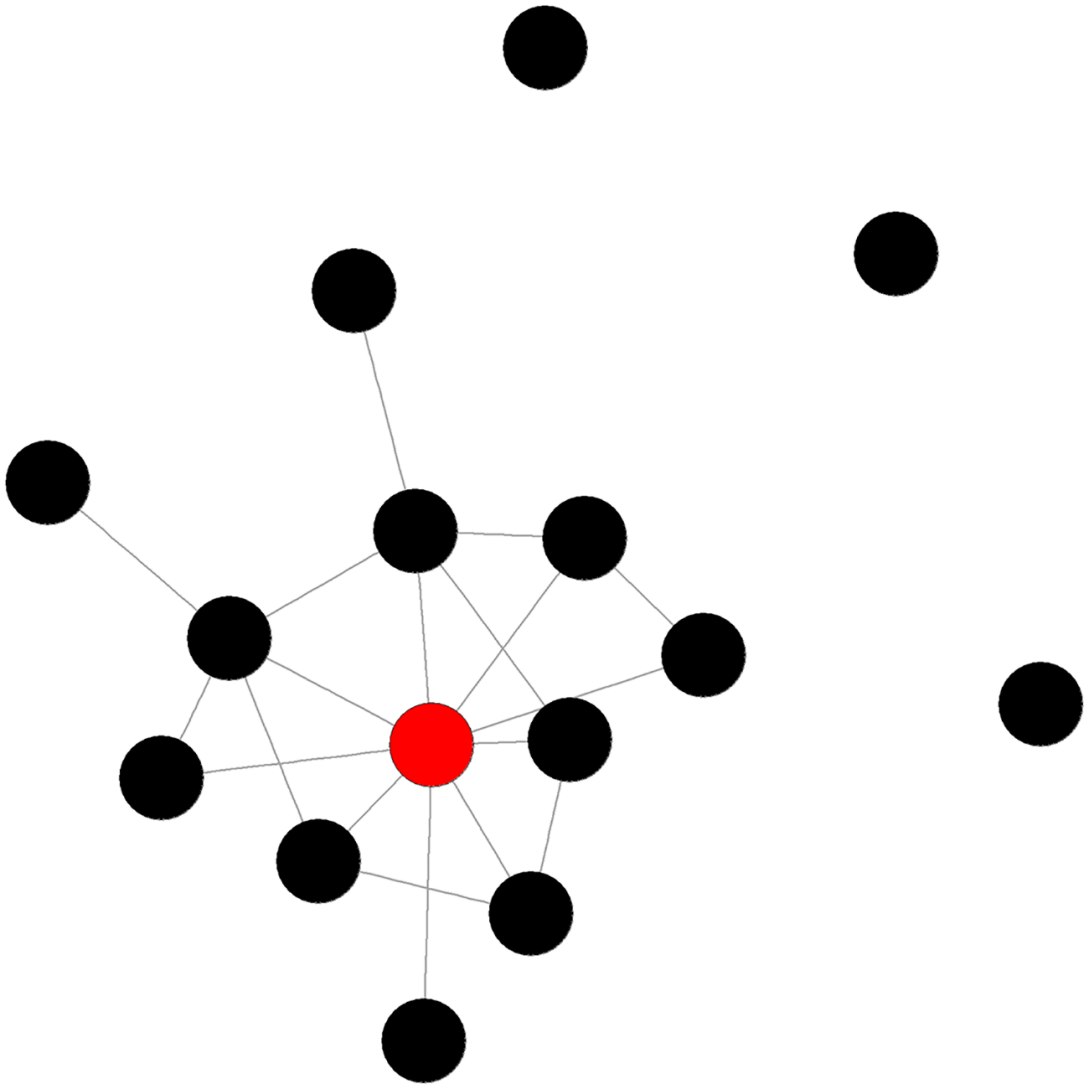
Example of a pig fighting network with high eigenvector centralisation. Individual with highest eigencentrality highlighted in red.

**Fig 4:**
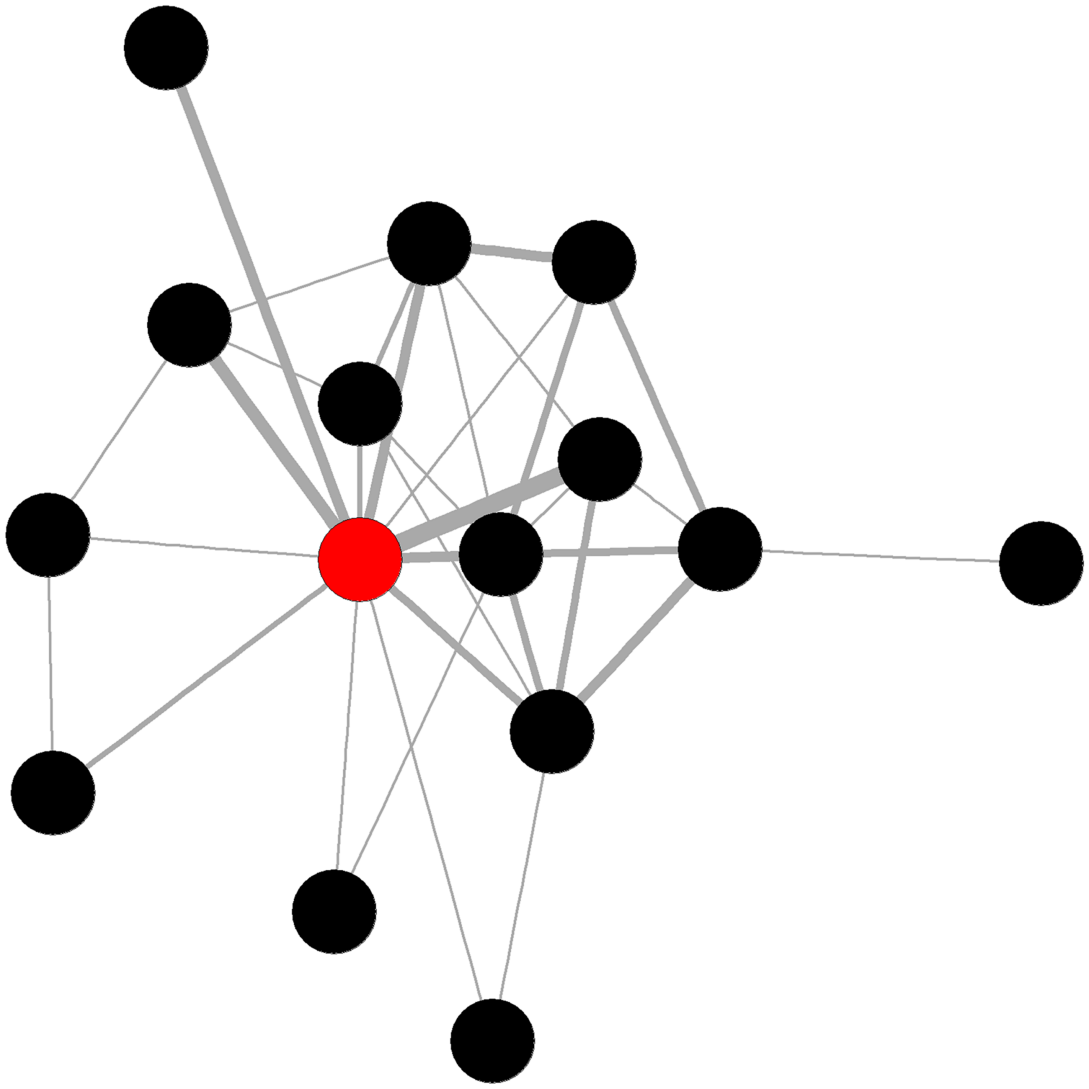
Example of a pig fighting network with high combined degree centralisation. An individual engaging in disproportionately more aggression (high degree) than the remaining pen mates is highlighted in red. Thicker edges represent frequency of interactions.

No network trait for posterior or total lesions at 24hr-PM significantly improved upon the partial model. At 24hr-PM fighting eigenvector centralisation (see Fig 3) was significantly negatively associated with lesions in the anterior region of the body (*F*_*1*, *58*_ = 11.24, *p* = 0.001). Pens showing high eigenvector centralisation tended to display highly localised aggression, with few aggressive individuals that engaged in many fights and also fought amongst themselves but with low connectivity amongst the remaining animals. Pens with high combined degree centralisation (See Fig 4) had significantly more lesions in the central (*F*_*1*, *58*_ = 4.52, *p* = 0.038) area of the body. Both eigenvector and combined degree centralisation improved upon the partial model (RMSE, AIC, and R^2^) (see Table 3).

**Table 3:** Model fit statistics.

	Trait		Estimate (SE)		RMSE			R2			AIC		
Lesion location	SNA	Dyadic	SNA	Dyadic	Partial	SNA	Dyadic	Partial	SNA	Dyadic	Partial	SNA	Dyadic
**24hr-PM**													
Anterior	Fighting eigenvector	Average fight duration	-33.87 (10.10)**	12.97 (3.41)***	5.49	5.03	4.92	0.33	0.44	0.46	432.0	415.1	414.6
Central	Combined degree centrality	-	4.84 (2.27)*	-	4.23	4.07	-	0.39	0.43	-	400.9	393.0	-
Posterior		Injurious fighting	-	-23.65 (10.19)*	2.27	-	2.16	0.49	-	0.54	302.0	-	290.3
Total	-	Average fight duration	-	15.90 (6.51)*	9.85	-	9.38	0.44	-	0.49	500.8	-	489.5
**3wk-PM**													
Anterior	Size of largest fighting clique	-	-0.67 (0.30)*		1.59	1.52	-	0.50	0.54	-	285.4	281.1	-
Central	Size of largest fighting clique	-	-1.18 (0.36)**		2.00	1.84	-	0.41	0.51	-	312.9	303.0	-
Posterior	Fighting betweenness	-	3.89 (1.79)*		1.00	0.96	-	0.61	0.64	-	231.4	223.8	-
Total	Size of largest fighting clique	-	-2.08 (0.76)**		4.41	3.89	-	0.50	0.55	-	398.5	390.1	-

Network properties and dyadic traits that were found to significantly improve model fit (p<0.05) are presented under ‘SNA’ and ‘Dyadic’. Model fit for fixed effects only are presented under ‘Partial’. In all trait models the number of aggressive interactions per pen was included. Blank cells indicate that no dyadic or network trait was found to significantly improve upon the partial model fit. Asterisks under estimate (SE) indicate level of significance.

*p*<0.05 * *p*<0.01** *p*<0.001***

At 24hr-PM, dyadic traits offered a better model fit for lesions than network properties in all body regions, apart from the central-region where no dyadic trait was found to improve upon the partial model.

Anterior lesions were positively associated with the average duration of fighting and bullying (*F*_*1*, *58*_ = 14.45, *p*<0.001), whereas posterior lesions were negatively associated with the proportion of fights that occurred in the pen that were classified as highly injurious (*F*_*1*, *58*_ = 5.38, *p* = 0.024). Total body lesions were positively associated with the average duration of all fighting and bullying behaviour (*F*_*1*, *58*_ = 5.97, *p* = 0.018).

**Fig 5:**
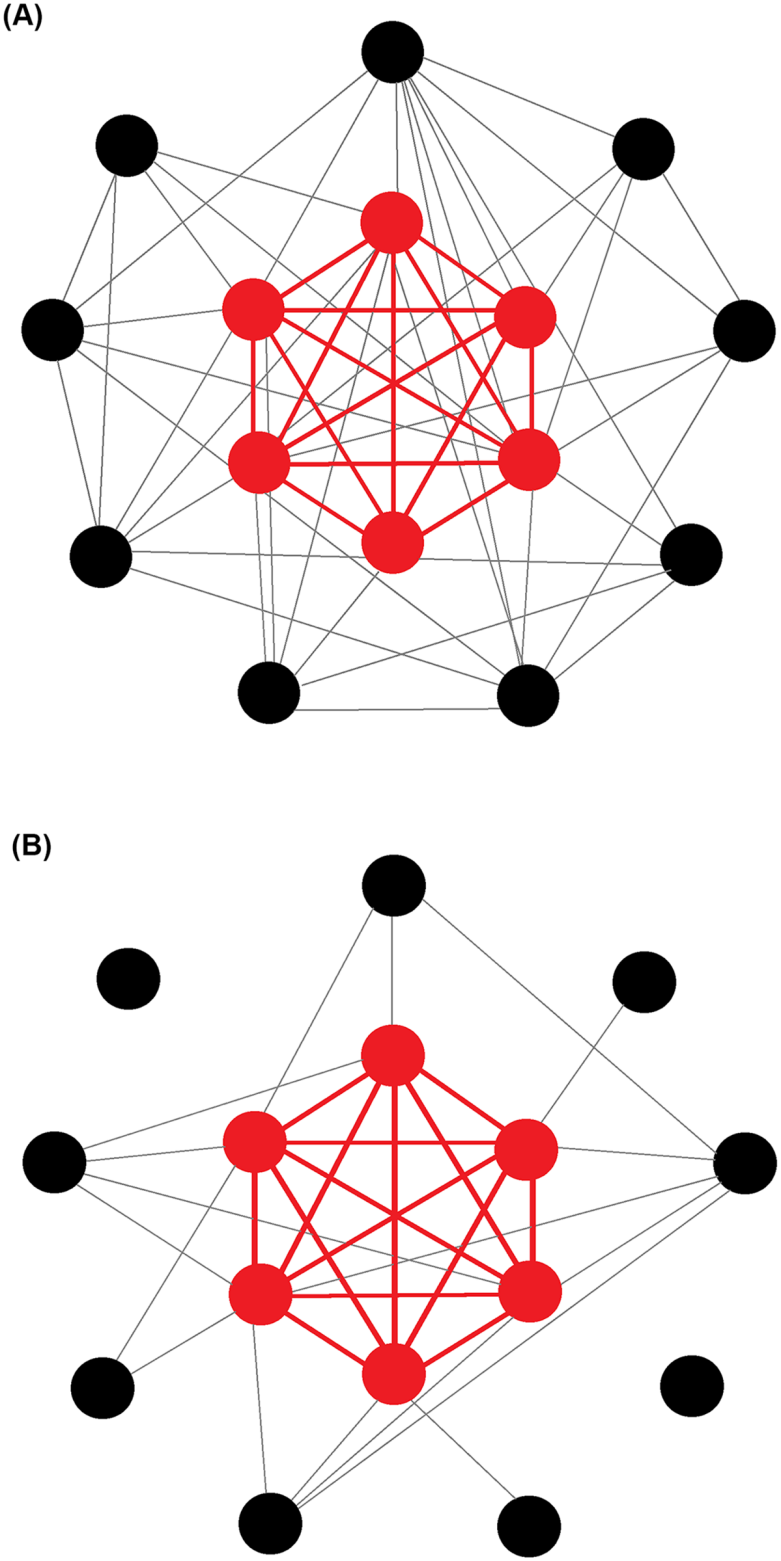
A) Example of a pig fighting network with a high number of aggressive interactions with a six animal clique. B) Example of a network with a lower number of aggressive interactions with a six animal clique. Cliques are highlighted in red for emphasis. Regardless of the difference in the number of interactions in each network, the presence of a clique is a strong predictor of the injuries the pen will have at a later date (3wk-PM).

In stable groups (3wk-PM), no dyadic traits offered any significant improvements upon the partial model in predicting lesions. The size of the largest fighting clique (see Fig 5) was found to provide the best single network trait model, revealing a strong negative association with lesions in three out of the four body regions at 3wk-PM (anterior: *F*_*1*,*58*_ = 5.02, *p* = 0.029, central: *F*_*1*,*58*_ = 10.77, *p* = 0.002, total: *F*_*1*,*58*_ = 7.47, *p* = 0.008). This indicates that pens that contained larger cliques during the 24hr period after introduction had lower rates of aggression related injuries at 3wk-PM.

**Fig 6:**
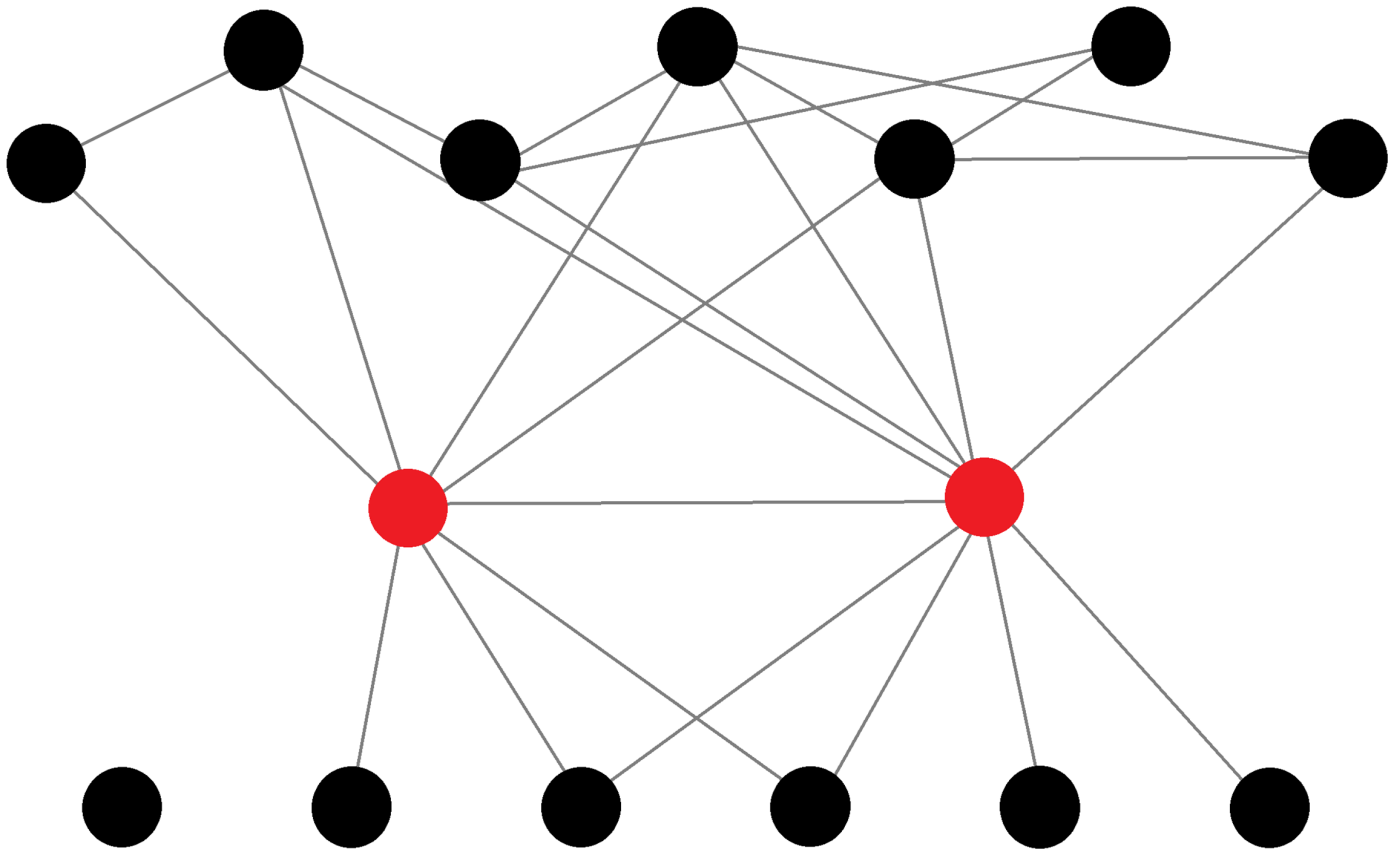
Example of a pig fighting network with high betweenness centralisation. Highly central individuals highlighted in red for emphasis.

Fighting betweenness centralisation (see Fig 6) had a positive association with posterior lesions 3wk-PM, indicating that pens that contained disjointed sub-groups connected by only a small number of animals when the group was formed, experience more lesions at the later time point. Fighting betweenness provided the best model for posterior lesions 3wk-PM (*F*_*1*,*58*_ = 4.74, *p*<0.034).

### Correlation between network and dyadic traits

The correlation coefficients between network properties and dyadic traits ranged from *r*_s_ -0.46–0.54 (a complete correlation matrix is available in supplementary material S4).

The only model where a network property and a dyadic trait were both found to be significant predictors was for anterior lesions at 24hr-PM (see Table 3). The significant predictors, eigenvector centralisation and average duration of fighting, were inversely associated, although this relationship was not significant (*r*_s =_ -0.21, *p* = 0.07).

For lesions at 3wk-PM, the network properties fighting clique size and betweenness centralisation were found to be significant predictors of lesions, and were significantly inversely related (*r*_s =_ -0.35, *p*<0.01).

### Permutations

**Fig 7:**
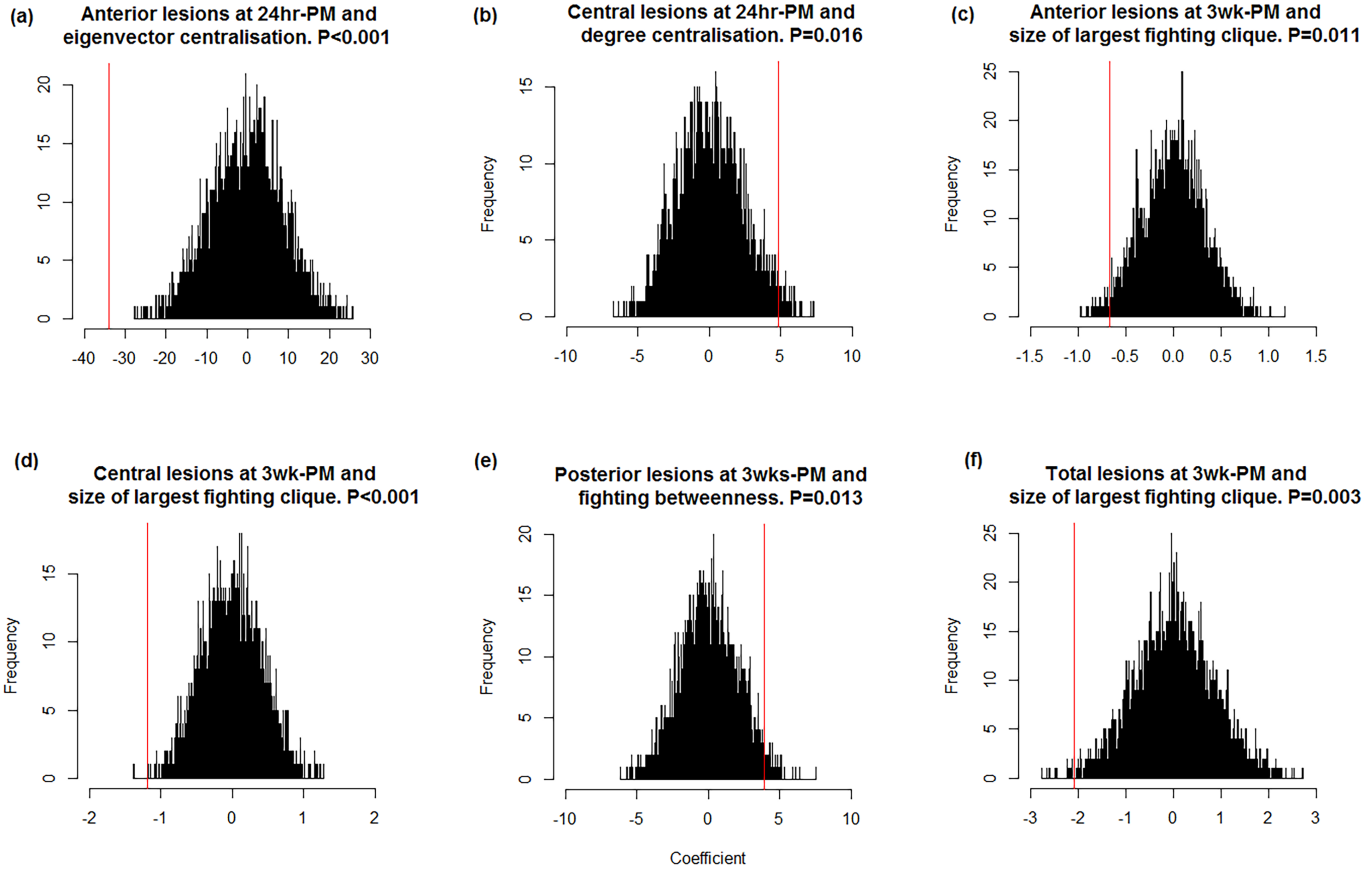
Coefficient frequency distributions from lesion models containing permuted network properties and the coefficient from the observed network models (in red). The red line represents the coefficient from the observed network properties. The observed value is considered to be significant if fewer than 2.5% of permuted values are greater than the observed value, or 97.5% of the permuted values are greater than the observed [44].

The permutations support the findings of the GLMM, and demonstrate that the network properties identified by the stepwise regression account for a significant proportion of the remaining variation present in pen level lesion scores (see Fig 7).

## Discussion

Network analysis is fast becoming a common approach to investigate the relationships between individual behaviour and population level functioning [16,17,21,23]. In this study we quantified the social network properties of multiple groups of pigs, with the objective of investigating whether group-level network properties can be used to predict subsequent amounts of pen levels injury at two time points resulting from aggressive interactions, and provide novel insights not captured by dyadic interactions.

Whilst network properties did not offer a model fit improvement compared to dyadic traits for predicting the injury caused by initial aggression associated with the establishment of dominance relationships (24hr-PM), network properties (betweenness centralisation and clique size) did provide predictive value of long term injury (3wk-PM), while dyadic traits did not.

Prior research has suggested that low levels of aggression upon introduction can lead to uncertain dominance relationships and chronic aggression [12]. However, our findings suggest that rather than the number of fights, it is the number of animals that are part of a fully connected subgroup (clique) that is a more important determinant of low chronic aggression. By controlling for the number of aggressive interactions per pen we were able to distinguish between the effect of number of fights and the different network properties that result from these aggressive interactions (see Fig 5). Our findings suggest that fights that form large cliques at 24hr-PM are more effective at decreasing chronic aggression than the same number of fights that do not form large cliques. As all animals in a clique have fought each other, clique members may form better established dominance relationships than non-clique members. This would explain the low level of aggression at 3wk-PM. Furthermore, as clique sizes did not exceed 47% of pen members, this suggests that a central group with established dominance relationships is sufficient to significantly reduce lesions at a pen level, without all group members needing to be involved directly.

Additionally, cliques suggest that in certain pens aggressive animals fight amongst themselves, whereas less aggressive animals are able to avoid engaging entirely. While it is expected that animals that do not engage in aggression at 24hr-PM may engage at a later date [12], the fact that a large clique size significantly reduced lesions to the anterior region suggests that the remainder of the pen did not engaging in fighting at 3wk-PM. However, analysing the individual lesion scores would be required in order to verify this.

Pens exhibiting high betweenness centralisation at 24hr-PM were at greater risk of high levels of posterior lesions at 3wk-PM than pens with lower centralisation. Posterior lesions are predominantly associated with receipt of bullying behaviour, as a fleeing animal turns away and the attacking animal inflicts injuries to the posterior portion of the body [12]. Therefore it is interesting that a fighting network property (that excluded all bullying behaviour) provided a strong model fit for lesions associated with receipt of bullying 3wk-PM. Once dominance relationships have been established, the maintenance of these relationships is usually achieved by the delivery of bullying to subordinate animals. It is possible that pens presenting high betweenness centralisation contain highly aggressive individuals that engage in excessive bouts of bullying to maintain their position, and cause elevated levels of posterior injuries to the remaining pen mates. Alternatively, the division present in pens with high betweenness centralisation indicates that there is a lack of direct contact between certain groups of animals. If insufficient interaction occurs in order to develop lasting dominance relationships throughout the pen, continued aggression may persist due to uncertain social positions within the group. Whilst network analysis has revealed network properties that predict chronic aggression and injury rates in newly mixed pigs, it has not quantified the costs and benefits to the individuals that have central roles in such networks. It is possible that the majority of the pen displays a form of aggression ‘avoidance’ [13] and are the ones responsible for the poor connectivity between groups, and the high betweenness individuals act as the connectors that improve the cohesiveness of a group that would otherwise be even more poorly connected [34]. Likewise, pens with a large clique may represent a highly aggressive subsection of a pen, and non-clique members are simply representative of animals able to avoid both acute and chronic aggression. Temporal analysis examining the process of network formation could help to understand how these central individuals assume this position. Comparing the injuries of central individuals at 24hr-PM and 3wk-PM to their pen-members may also reveal the long and short terms costs and benefits of occupying a position of high centrality.

The results have revealed that fighting and bullying networks significantly differ in their contribution to chronic aggression. This raises the question of whether the remaining variance in lesions could be further explained by the inclusion of other social behaviours aside from aggression. For example, in rhesus macaque groups individuals that were responsible for the maintenance of social network stability in aggression networks were also key players in grooming behaviour networks [45], suggesting a variety of social behaviours relate to conflict management. Pigs have a complex range of negative and pro-social interactions and behaviours, which could play a role in resolution of conflict [46]. Future research should consider the inclusion of additional social behaviour, as this may provide a more complete understanding of an animal’s social standing and could improve the predictive value of network properties.

## Conclusion

Our findings provide further support that network properties have the potential to outperform dyadic traits in predicting long term social outcomes [20]. Our results suggest that division in newly mixed groups of pigs is likely to lead to prolonged chronic aggression and elevated injury rates, whereas pens with large cliques (~47% of the pen members) are likely to have significantly fewer injuries in stable groups. We suggest that these network properties indicate that divided networks represent poorly established dominance relationships at a pen level, and large cliques indicate that a sufficient proportion of the group has established their social position and thus reduces the need for prolonged aggression.

## Supporting information

S1 File Freeman centralisation. (DOCX)

S1 Table Network properties estimated from the behavioural networks and entered in the stepwise regression. Separate networks were created using fighting only and bullying only and these behaviours combined. Directed versions of degree and closeness were applied to bullying networks only.
(DOCX)

S2 Table Summary of key terms and network properties. (DOCX)

S3 Table Descriptive statistics of network properties in fighting, bullying and combined networks. (DOCX)

S4 Table Spearman rank correlation of dyadic and network properties. (DOCX)
