# Supplementary material for: Social network properties predict chronic aggression in commercial pig systems
Source: PLoS One. 2018 Oct 4;13(10):e0205122. doi: 10.1371/journal.pone.0205122 (PMC6171926; doi:10.1371/journal.pone.0205122)
Supplement: S1 File — (DOCX) [file pone.0205122.s001.docx]

**S1. Freeman centralisation**

The group-level centrality score *C(G)* for a group (pen) *G* was calculated as:
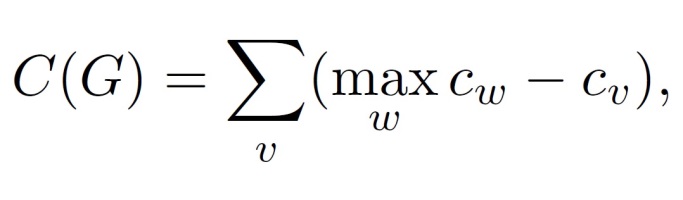


where max *c_w_* refers to the maximum centrality score of all group members *w* and the sum of individual centrality scores *c_v_* is taken over all group members.
