# Supplementary material for: Social network properties predict chronic aggression in commercial pig systems
Source: PLoS One. 2018 Oct 4;13(10):e0205122. doi: 10.1371/journal.pone.0205122 (PMC6171926; doi:10.1371/journal.pone.0205122)
Supplement: S1 Table — Separate networks were created using fighting only and bullying only and these behaviours combined. Directed versions of degree and closeness were applied to bullying networks only. (DOCX) [file pone.0205122.s002.docx]

Table S1. Network properties estimated from the behavioural networks and entered in the stepwise regression.

| **Network properties** | **Behavioural Networks** | | |
| --- | --- | --- | --- |
|  | **Bully** | **Fight** | **Combined** |
| Clustering-coefficient | x | x | x |
| Largest Clique | x | x | x |
| Degree | x | x | x |
| Eigenvector | x | x | x |
| Betweenness | x | x | x |
| Closeness | x | x | x |
| In-degree | x | - | - |
| Out-degree | x | - | - |
| In-closeness | x | - | - |
| Out-closeness | x | - | - |

Separate networks were created using fighting only and bullying only and these behaviours combined. Directed versions of degree and closeness were applied to bullying networks only.
