# Supplementary material for: Social network properties predict chronic aggression in commercial pig systems
Source: PLoS One. 2018 Oct 4;13(10):e0205122. doi: 10.1371/journal.pone.0205122 (PMC6171926; doi:10.1371/journal.pone.0205122)
Supplement: S2 Table — (DOCX) [file pone.0205122.s003.docx]

**Table S2. Summary of key terms and network properties.**

| **Terminology** | **Definition** |
| --- | --- |
| Graph | A set of nodes and the set of relationships between them. |
| Node | An individual in a network with known interactions to other individuals within the network |
| Behavioural network | Each pen has a network comprised of either only bullying or fighting, or both behaviours combined. |
| Dyadic traits | Behavioural traits based on dyadic interactions that do not take account of third party interactions. |
| Network traits | Umbrella term to refer to all quantitative descriptors of network properties derived from network measures. |
| Edge | The interaction between two nodes |
| Edge direction | Edges can be directed based upon the nature of the interaction. The edge direction travels from the ‘sender’ towards the ‘receiver’. |
| Path length | A path is the number of edges that lie between two nodes. |
| Individual measures | An individual measure quantifies the position of individual nodes in a network |
| Global measures | A global measure quantifies the network as a whole. |
| Centrality | A category of approaches which are used to identify which nodes occupy a ‘central’ or an influential position within a network. |
| Centralisation | Freeman’s equation of centralisation describes the inequality in centrality within a network. |
| **Definitions of network traits used in this paper** | |
| Degree  centralisation | A centralised network is one in which one individual has considerably more direct connections than all others in the network. Degree can also be calculated as ‘in-degree’ or ‘out-degree’. Directed degree centralisation informs us whether certain individuals within a network receive or give substantially more interactions than other individuals in the network. |
| Eigenvector centralisation | Eigenvector centrality takes account of the number and quality of the indirect connections an animal has. A centralised network is one in which highly connected individuals tend to interact with each other. |
| Betweenness centralisation | Betweenness calculates the extent to which a node is present on the shortest path (geodesic) between other nodes. A centralised network one or a few individuals that interact with nodes that they themselves do not interact directly. |
| Closeness centralisation | A node that has high closeness has a short distance to all other nodes in the network.  A centralised network has one or a few individuals with shorter paths to all individuals in the pen than the rest of the animals. |
| Largest clique | In this paper we present the largest clique as the *number of individuals* that belong to the largest clique. |
| Clique | A group of fully connected nodes. |
| Clustering coefficient | The probability that a node’s connections are also connected. E.g A is connected to B and C. If B and C are also connected, this leads to a higher clustering co-efficient. |
