# Supplementary material for: Social network properties predict chronic aggression in commercial pig systems
Source: PLoS One. 2018 Oct 4;13(10):e0205122. doi: 10.1371/journal.pone.0205122 (PMC6171926; doi:10.1371/journal.pone.0205122)
Supplement: S3 Table — (DOCX) [file pone.0205122.s004.docx]

**Table S3. Descriptive statistics of network properties in fighting, bullying and combined networks.**

| **Network Property** | **Behavioural network** | **Median** | **Minimum** | **Maximum** |
| --- | --- | --- | --- | --- |
| Largest clique | Fight | 4.00 | 3.00 | 7.00 |
|  | Bully | 4.00 | 3.00 | 7.00 |
|  | Combined | 5.00 | 4.00 | 8.00 |
| Clustering Co-efficient | Fight | 0.46 | 0.14 | 0.68 |
|  | Bully | 0.39 | 0.08 | 0.64 |
|  | Combined | 0.58 | 0.36 | 0.80 |
| Closeness | Fight | 0.16 | 0.07 | 0.63 |
|  | Bully | 0.34 | 0.07 | 0.80 |
|  | Bully (In) | 0.06 | 0.01 | 0.35 |
|  | Bully (Out) | 0.24 | 0.04 | 0.75 |
|  | Combined | 0.39 | 0.13 | 0.77 |
| Betweenness | Fight | 0.15 | 0.06 | 0.41 |
|  | Bully | 0.21 | 0.08 | 0.62 |
|  | Combined | 0.14 | 0.01 | 0.53 |
| Degree | Fight | 0.30 | 0.17 | 0.54 |
|  | Bully | 0.35 | 0.12 | 0.64 |
|  | Bully (In) | 0.20 | 0.09 | 0.42 |
|  | Bully (Out) | 0.39 | 0.12 | 0.78 |
|  | Combined | 0.33 | 0.14 | 0.61 |
| Eigenvector | Fight | 0.51 | 0.32 | 0.74 |
|  | Bully | 0.52 | 0.30 | 0.76 |
|  | Combined | 0.40 | 0.16 | 0.60 |
