# Supplementary material for: Social network properties predict chronic aggression in commercial pig systems
Source: PLoS One. 2018 Oct 4;13(10):e0205122. doi: 10.1371/journal.pone.0205122 (PMC6171926; doi:10.1371/journal.pone.0205122)
Supplement: S4 Table — (DOCX) [file pone.0205122.s005.docx]

**Table S4. Spearman rank correlation of dyadic and network properties**

|  | Combined  degree | Fighting eigenvector | Fighting betweenness | Size of the largest clique | Average duration of bullying and fights | Proportion of injurious fighting |
| --- | --- | --- | --- | --- | --- | --- |
| Combined degree |  |  |  |  |  |  |
| Fighting eigenvector | 0.17 |  |  |  |  |  |
| Fighting betweenness | 0.21 | 0.54** |  |  |  |  |
| Size of the largest clique | -0.17 | -0.46** | -0.35* |  |  |  |
| Average duration of bullying and fights | -0.27* | -0.21 | -0.13 | 0.10 |  |  |
| Proportion of injurious fighting | -0.15 | -0.24* | -0.06 | 0.26* | 0.30* |  |
